# Brain region-specific gene expression profiles in freshly isolated rat microglia

**DOI:** 10.3389/fncel.2015.00084

**Published:** 2015-03-12

**Authors:** Karlijn J. Doorn, John J. P. Brevé, Benjamin Drukarch, Hendrikus W. Boddeke, Inge Huitinga, Paul J. Lucassen, Anne-Marie van Dam

**Affiliations:** ^1^ Department Structural and Functional Plasticity of the Nervous System, Center for Neuroscience, Swammerdam Institute for Life Sciences, University of AmsterdamAmsterdam, Netherlands; ^2^ Neuroscience Campus Amsterdam, Department Anatomy and Neurosciences, VU University Medical CenterAmsterdam, Netherlands; ^3^ Section Medical Physiology, Department of Neuroscience, University Medical Centre GroningenGroningen, Netherlands; ^4^ Neuroimmunology Group, Netherlands Institute for Neuroscience, Institute of the Royal Netherlands Academy of Arts and SciencesAmsterdam, Netherlands

**Keywords:** Parkinson’s disease, substantia nigra, hippocampus, olfactory bulb, microglia, FACS

## Abstract

Microglia are important cells in the brain that can acquire different morphological and functional phenotypes dependent on the local situation they encounter. Knowledge on the region-specific gene signature of microglia may hold valuable clues for microglial functioning in health and disease, e.g., Parkinson’s disease (PD) in which microglial phenotypes differ between affected brain regions. Therefore, we here investigated whether regional differences exist in gene expression profiles of microglia that are isolated from healthy rat brain regions relevant for PD. We used an optimized isolation protocol based on a rapid isolation of microglia from discrete rat gray matter regions using density gradients and fluorescent-activated cell sorting. Application of the present protocol followed by gene expression analysis enabled us to identify subtle differences in region-specific microglial expression profiles and show that the genetic profile of microglia already differs between different brain regions when studied under control conditions. As such, these novel findings imply that brain region-specific microglial gene expression profiles exist that may contribute to the region-specific differences in microglia responsivity during disease conditions, such as seen in, e.g., PD.

## Introduction

Microglia are the primary, innate immune cells of the central nervous system (CNS) responsible for safeguarding and maintenance of brain homeostasis. By constantly surveying their microenvironment, microglia can quickly respond to a disturbed homeostasis caused, by e.g., pathogens or injury. Under such conditions, microglia undergo typical morphological and functional alterations and become engaged in processes like phagocytosis, cytokine production, antigen presentation and/or cell proliferation (Nimmerjahn et al., 2005; Hanisch and Kettenmann, 2007; Kettenmann et al., 2011; Doorn et al., 2014a).

Although prior studies have suggested that microglial activation is largely detrimental in the context of homeostatic brain disturbances, it is now well accepted that microglia can also benefit CNS function and provide neuroprotection, shape neuronal circuits and promote axonal regeneration (Ekdahl et al., 2003; Walter and Neumann, 2009; Welser et al., 2010; Czeh et al., 2011; Wake et al., 2013). In addition, while knowledge on general microglial morphology and function is well established, only recently has it become clear that microglial activity may be region-specific. Hence, previous views on microglia as a rather uniform cell population present throughout the brain, have now been replaced by the concept that these cells can acquire specific phenotypes depending on their region-specific environment (Lucin and Wyss-Coray, 2009; Ransohoff and Perry, 2009; Olah et al., 2011).

Besides regional differences in microglial density, which are positively correlated with, e.g., differential sensitivity to LPS-induced neurotoxicity (Kim et al., 2000), microglia display a region-dependent diversity in their surface markers expression (Lawson et al., 1990; Mittelbronn et al., 2001; De Haas et al., 2008; Buschmann et al., 2012). In addition, injection of interferon gamma (IFN-γ) into the brain caused a significant upregulation of major histocompatibility complex II (MHC II) molecules by microglia in the brainstem but not in the HC (Peng et al., 1998; Olah et al., 2011). Although such overt region-specific microglial responsiveness could be a mere consequence of differences in the local microenvironment, microglia isolated from different brain regions also present a distinct inflammatory profile at basal conditions as well as following endotoxin stimulation *in vitro*. This suggest that at least some of these traits are inherent to the microglia cells themselves, e.g., M1 or M2 type microglia (Ren et al., 1999;Xie et al., 2003).

Microglial diversity has also raised interest in their role in various neurodegenerative diseases, including PD (McGeer et al., 1988; Perry et al., 2010; Halliday and Stevens, 2011; Doorn et al., 2012; Hirsch et al., 2012). Recent data have shown that α-synuclein pathology spreads throughout the brain and affects several brain regions in PD patients (Braak et al., 2004), including the SN, OB, brainstem, limbic system and neocortex, in a disease-stage dependent manner (Braak and Braak, 2000; Amino et al., 2005; Orimo et al., 2007; Dickson et al., 2009; Jellinger, 2011). Microglia have been proposed to contribute to this differential, and evolving pattern of neuropathology that may underlie at least some of the motor and non-motor symptoms in PD (Doorn et al., 2014b). Besides the identification of activated microglia in the SN, i.e., a classical neuropathological PD site, microglial activation also occurs in non-dopaminergic regions like the OB and HC, notably regions where no or little neuronal loss occurs in PD (Imamura et al., 2003; McGeer and McGeer, 2008; Doorn et al., 2013, 2014b). In addition, we recently observed expression of microglial Toll-like receptor-2 (TLR2), that is involved in α-synuclein-mediated microglial activation, to be different between the HC and SN of PD patients (Kim et al., 2013; Doorn et al., 2014b), consistent with the concept of region-specific microglial phenotypes in human tissue.

The presence and profile of microglial surface markers not only differ between different brain regions in healthy control mice, also a differential responsiveness to various stimuli has been documented (Peng et al., 1998; Vroon et al., 2007; De Haas et al., 2008; Olah et al., 2011; Watson et al., 2012). Prior to studying region-specific differences in microglial expression profiles in a disease model, we here study whether gene expression profiles of rat microglia are already region-specific under baseline conditions. This is of importance as this expression profile might predict microglial responsivity, and hence differential pathological outcomes under challenging conditions. To this end, we used an optimized protocol to isolate microglia under baseline conditions from small amounts of tissue based on density gradients and fluorescent-activated cell sorting (FACS), which subsequently allowed the analysis of (inflammatory) gene expression profiles in pure microglia derived from brain regions of relevance for PD, e.g., the SN or other regions relevant for PD.

## Materials and Methods

### Animals

All experimental procedures were performed and carried out with approval of the animal ethical committee of the VU University Medical Center. In each independent set of experiments (*n* = 3), two adult male Wistar rats (250 g, Harlan) were sacrificed and the brain was rapidly removed and kept in GKN/BSA buffer (see below) on ice.

### Acute Microglia Isolation

#### Percoll Solutions

Percoll (GE healthcare Biosciences, Uppsala, Sweden) was diluted 1:10 in sterile 10x PBS to yield 100% isotonic Percoll. This isotonic Percoll was diluted in GKN/BSA buffer (GKN 0.8 g/l NaCl, 0.4 g/l KCl, 3.6 g/l Na_2_HPO_4_.12H_2_0, 0.8 g/l NaH_2_PO_4_, 2 g/l d-(+)-glucose, 0.3% BSA; pH 7.4, 4^∘^C) to yield 75% and 50% isotonic Percoll solutions.

#### Tissue Processing

**FIGURE 1:**
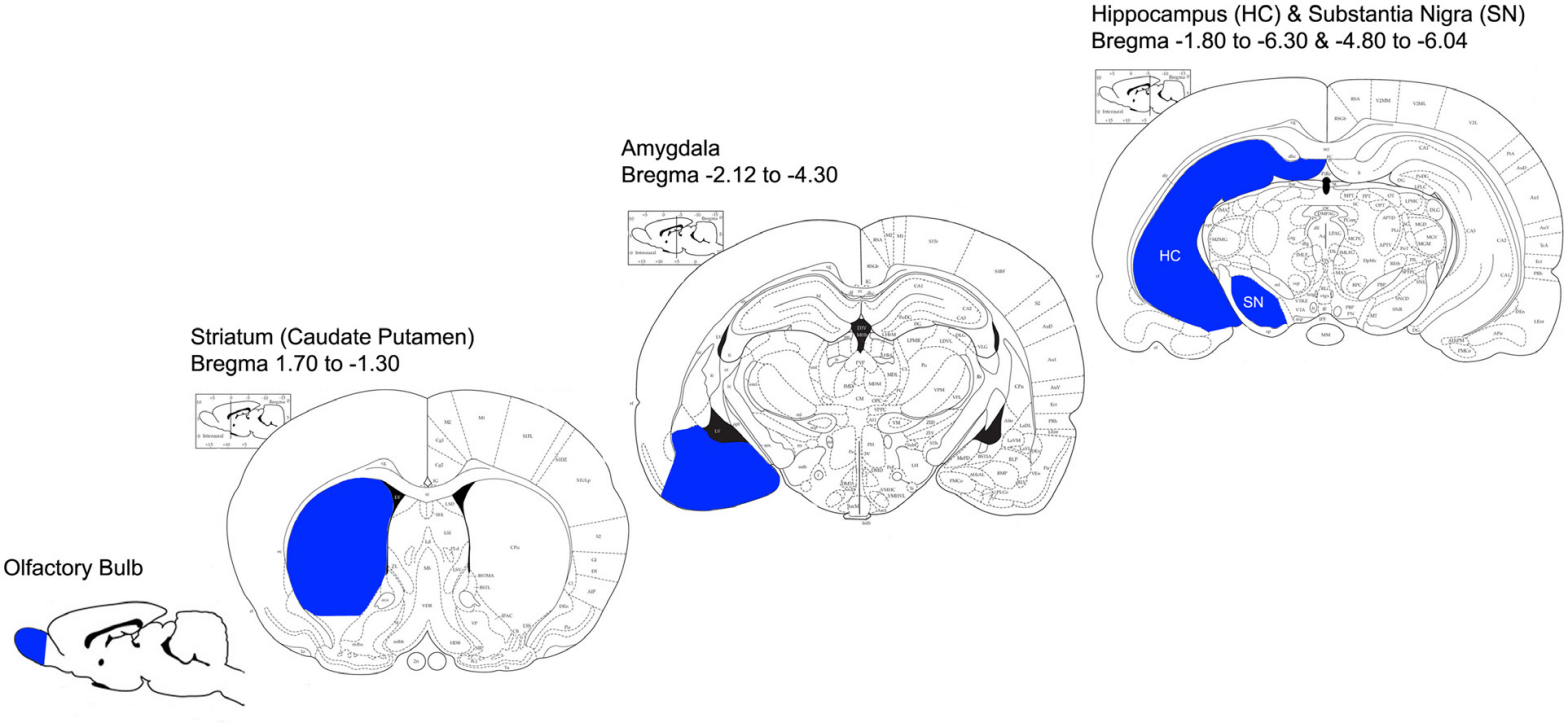
Anatomical representation of dissected brain regions (adapted from the Rat Brain Atlas; https://gaidi.ca/rat-brain-atlas/). Brain regions that were dissected for microglia isolation are indicated in blue; OB, STR, AM, HC and SN. Bregma coordinates indicate the anterior-posterior range of each region that was dissected.

All procedures were carried out on ice. After removal of the meninges, OB, amygdala (AM), HC, striatum (STR) and SN were rapidly dissected, and placed separately in a plastic Petri dish containing 2 ml of ice-cold GKN/BSA buffer (see **Figures 1** for information on anatomical location of dissected regions). Identical regions from 2 rats were pooled per experiment to increase microglial yield. Tissue was minced with a razor blade and transferred to a 70 μm pore size strainer (BD Biosciences, Erembodegem, Belgium) on top of a 50 ml conical tube (Greiner Bio-One). Tissue was then gently dissociated and mashed through the strainer to reach a single cell suspension. An additional 30 ml GKN/BSA was added to the tube containing the cell suspension, and then the tubes were centrifuged for 10 min at 300x *g* at 4^∘^C (Hettich, Tuttlingen, Germany).

#### Density Gradient Centrifugation

The supernatant was discarded and the remaining cell pellet was resuspended in 1 ml 50% Percoll and transferred to a new 15 ml polystyrene tube after which an additional 7 ml 50% Percoll was added. Then 4 ml of 75% Percoll was gently layered underneath the 50% Percoll layer and subsequently 3 ml GKN/BSA buffer was layered on top of the 50% Percoll layer using a Pasteur pipette. The density gradient was centrifuged at 1300x *g* (Hettich, Tuttlingen, Germany) for 30 min at 4^∘^C.

#### Microglia Collection

Two distinct layers were apparent after centrifugation. The top layer between the GKN/BSA and 50% Percoll gradient consisted mainly of thick, viscous myelin. The lower layer at the interphase between the 50 and 75% Percoll phases appeared quite faint and contains highly enriched microglia. First, the top layer was carefully removed and then, using a new Pasteur pipette, the microglia containing interphase was aspirated and transferred to a 15 ml polystyrene tube. Cells were washed twice with 14 ml of GKN/BSA buffer and after adding another 14 ml of GKN/BSA buffer, the cells were centrifuged for 7 min at 300x *g* at 4^∘^C and the buffer was discarded.

### Fluorescence-Activated Cell Sorting (FACS)

#### Staining of Microglial Cells

Immediately after Percoll gradient separation and washing steps, cells were stained with the following antibodies: Pacific blue-labeled mouse anti CD11b (Serotec, MCA275PB; final dilution 1:30) or Alexa647-labeled mouse anti CD45 (Serotec, MCA 43A647; final dilution 1:30), including isotype controls (IgG2a, IgG1) to control for background staining. Briefly, cells per region were incubated for 30 min at 4^∘^C in antibody diluted in PBS and shielded from light. In addition, cells were incubated for 10 min with Sytox green nucleic acid stain (Molecular Probes, S7020; final dilution 1:500,000) to distinguish living from dead cells, shortly before flow cytometry. Subsequently, cells were rinsed twice in PBS, pelleted at 1200 rpm for 5 min at 4^∘^C and resuspended in 300 μl PBS before they were filtered over a 70 μm size strainer to obtain a single cell suspension ready for FACS analysis and sorting.

#### FACS Analysis and Sort of Microglia

Cell size, granularity, and fluorescence intensity were analyzed per brain region (*n* = 3) with a MoFlo fluorescent activated cell sorter (Beckman Coulter, Mijdrecht, Netherlands). Approximately 2x10^4^ events were counted and subsequently analyzed using Summit software version 4.3 (DAKO) to determine gates before cells were sorted. Microglia were defined as the percentage of all living, Sytox green negative, cells that showed CD11b^pos^/CD45^low^ expression (Sedgwick et al., 1991). Subsequently, for each brain region, the FACS sorted microglial cells were transferred into RNAse-free tubes and centrifuged for 5 min at 300x *g* (Hettich, Tuttlingen, Germany) at 4^∘^C. The supernatant was aspirated, and cells were lysed with 500 μl Trizol reagent (Invitrogen, Carlsbad, CA, USA) for 5 min at RT. Lysates were stored at -80^∘^C for subsequent RNA isolation, cDNA synthesis and qPCR analysis.

### RNA Isolation, cDNA Synthesis and Quantitative Real-Time PCR

Total RNA (*n* = 3/brain region) was isolated from sorted microglia per brain region using Trizol Reagent (Invitrogen, Carlsbad, CA, USA) according to the manufacturer’s instructions. Total RNA was further purified using the RNeasy MinElute Cleanup kit (Qiagen). RNA was reverse-transcribed into cDNA using the High-Capacity cDNA Reverse Transcription kit (Life Technologies), using 0.5 μg oligo-dT primers and according to the manufacturer’s instructions. For subsequent quantitative real-time PCR (qPCR), the Power SYBR Green Master Mix (Life Technologies) was used. Primers were purchased from Eurogentec (Maastricht, Netherlands) and qPCR was performed in MicroAmp Optical 96-well Reaction Plates (Applied Biosystems) on a StepOnePlus Real-Time PCR system (Applied Biosystems). The reaction mixture (20 μl) was composed of 1 × Power SYBR Green buffer (Applied Biosystems), 3.75 pmol of each primer (see **Table 1** for primer details), and 12.5 ng cDNA. The thermal cycling conditions were an initial 10 min at 95^∘^C followed by 40 cycles of 15 s at 95^∘^ C and 1 min at 60^∘^C. The specificity of the reaction was checked by means of melt curve analysis. The relative expression level of the target genes was determined by the LinRegPCR software (version 2014.3; website: http://www.hfrc.nl) using the following calculation N0 = Nq/E^Cq^ (N0 = target quantity, Nq = fluorescence treshold value, *E* = mean PCR efficiency per amplicon, Cq = treshold cycle (Ruijter et al., 2009), after which the value was normalized relative to glyceraldehyde-3-phosphate-dehydrogenase (GAPDH). We chose GAPDH as reference gene based on previous studies (Roy et al., 2006; Melief et al., 2012). Furthermore, no *in vivo* or *ex vivo* treatments have been performed with the cells that may change the expression of reference genes.

**Table 1:** Primer sequences used for q-PCR analysis.

Rat genes	Alignment sequence	Forward (5^′^→3^′^)	Reverse (5^′^→3^′^)	bp
GAPDH	NM_017008.3	GAACATCATCCCTGCATCCA	GCCAGTGAGCTTCCCGTTCA	79
GFAP	NM_017009.2	CAGACTTTCTCCAACCTCCAG	CTCCTGCTTCGAGTCCTTAATG	138
TNFα	NM_012675.3	CCACACCGTCAGCCGATT	TCCTTAGGGCAAGGGCTCTT	81
TLR2	NM_198769.2	GAGCATCGGCTGGAGGTCT	CTGGAGCTGCCATCACACAC	160
ITGAM	NM_012711.1	TTATTGGGGTGGGAAACGCCT	CTGGAGCTGGTTCCGAATGGT	137
AIF1	NM_017196.3	GCCTCATCGTCATCTCCCCA	AGGAAGTGCTTGTTGATCCCA	142
IL-1β	NM_031512.2	AAAGAAGAAGATGGAAAAGCGGTT	GGGAACTGTGCAGACTCAAACTC	81
CD68	NM_001031638.1	CTCATCATTGGCCTGGTCCT	GTTGATTGTCGTCTGCGGG	80
NOS2	NM_012611.3	AACTTGAGTGAGGAGCAGGTTGA	CGCACCGAAGATATCCTCATGA	81
MSR1	NM_0011939.1	AAAGGCAGGGAGGTCAGGATTTC	CCGCTACCATCAACCAGTCG	89
P2X_ 7_R	NM_001038839.1	ACCCTCAGTGTTCCATCTTCCG	TTCCTCCCTGAACTGCCACC	84


*GAPDH, glyceraldehyde-3-phosphate dehydrogenase; GFAP, glial fibrillary acidic protein; TNFα, tumor necrosis factor alpha; TLR2, toll-like receptor 2; ITGAM, integrin, alpha M (CD11b); AIF1, allograft inflammatory factor 1 (Iba-1); IL-1β, interleukin-1 beta; CD68, cluster of differentiation 68; NOS2, nitric oxide synthase 2; MSR1, macrophage scavenger receptor 1; P2X7R, P2X purinoreceptor 7 bp, amplicon size base pair.*

### Statistical Analysis

Statistical analyses were performed using the SPPS package version 20.0 (Statistical Product and Service Solutions, Chicago, IL, USA). The mean and SD of efficiency corrected and GAPDH normalized mRNA expression in microglia were calculated for each brain region. Since data was normally distributed, as determined by the Shapiro-Wilk test, within statistical analysis was performed using a paired sample *T*-test. Bonferroni corrections for multiple testing led to a *p*-value of 0.005 (alpha/number of tests: 0.05/10). However, since this may be too stringent for our analysis leading to type 2 errors (false negative), we indicated the results in our graphs as significant with *p*-value set at 0.05.

## Results

### FACS Sorted Microglia from Various Rat Brain Regions

**FIGURE 2:**
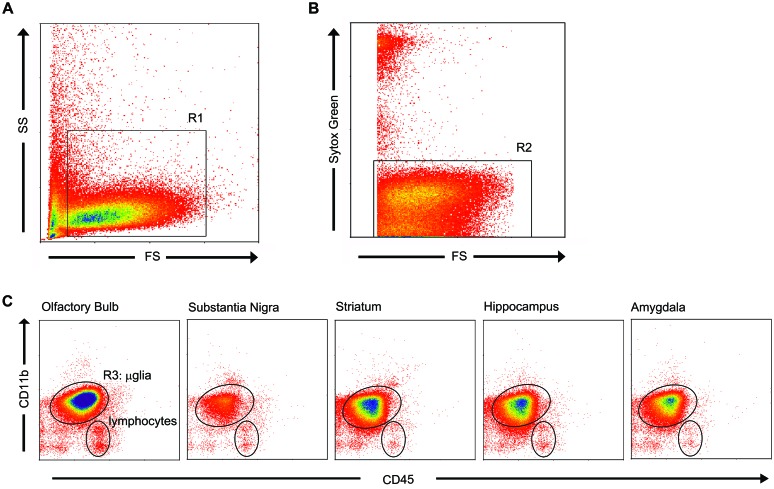
Flow cytometric analysis based on cell size, granularity and viability identified microglia with CD11b^**high**^/CD45^**pos**^ expression. **(A)** Microglia appeared homogeneous in cell size and granularity (R1). **(B)** Sytox green staining, a DNA-binding dye that is actively taken up by dying cells, distinguished the living cells (R2) from dead cells and debris. **(C)** Combined selection based on cell size, granularity, and viability (R1 and R2) resulted in a microglial purity of 92.4 ± 0.87% (mean ± SD), as identified by their characteristic CD11b^high^/CD45^pos^ expression. A small fraction of lymphocytes was present to within the viable cells. To obtain a 100% pure microglial population, microglia (R3) were sorted.

For the isolation of microglia, we optimized earlier developed protocols with respect to microglia survival and purity (Frank et al., 2006; De Haas et al., 2008; Melief et al., 2012). Upon flow cytometry, microglia were identified based on cell size, granularity (**Figures 2A**, R1) and viability by Sytox green nucleic acid staining (**Figures 2B**, R2). As it has been shown that in gray matter brain regions of untreated rodents, hardly any monocytes/macrophages can be observed (Lawson et al., 1990), we identified a large homogeneous CD11b^high^/CD45^pos^ cell population which we considered being only microglia, revealing a population of 90–95% of all viable cells (mean ± SD, **Figures 2C**; **Table 2**). A small amount (mean of 7%) of CD11b^low^/CD45^pos^cells (**Figures 2C**), especially observed in the OB, were identified as lymphocytes (Lacombe et al., 1997; Stirling and Yong, 2008). After sorting of the microglial cells, their amount ranged from 1.1 × 10^4^ microglial cells in the SN to 1.2 × 10^5^ microglial cells in the OB (**Table 2**). The CD11b^high^/CD45^pos^ expressing cells were then sorted to obtain a pure microglia population for further analysis (**Figures 2C**).

**Table 2:** Quantification of isolated microglia per brain region.

Brain regions	OB	SN	HC	STR	AM
% Pure microglia of viable cells (R2)	95	90	91	93	93
# Sorted microglial cells	1.2 × 10^5^	1.1 × 10^4^	6.5 × 10^4^	7.2 × 10^4^	4.3x101^4^


*#, number; %, percentage; OB, olfactory Bulb; SN, substantia nigra; HC, hippocampus; STR, striatum; AM, amygdale*

### Brain Region-Specific Gene Expression

**FIGURE 3:**
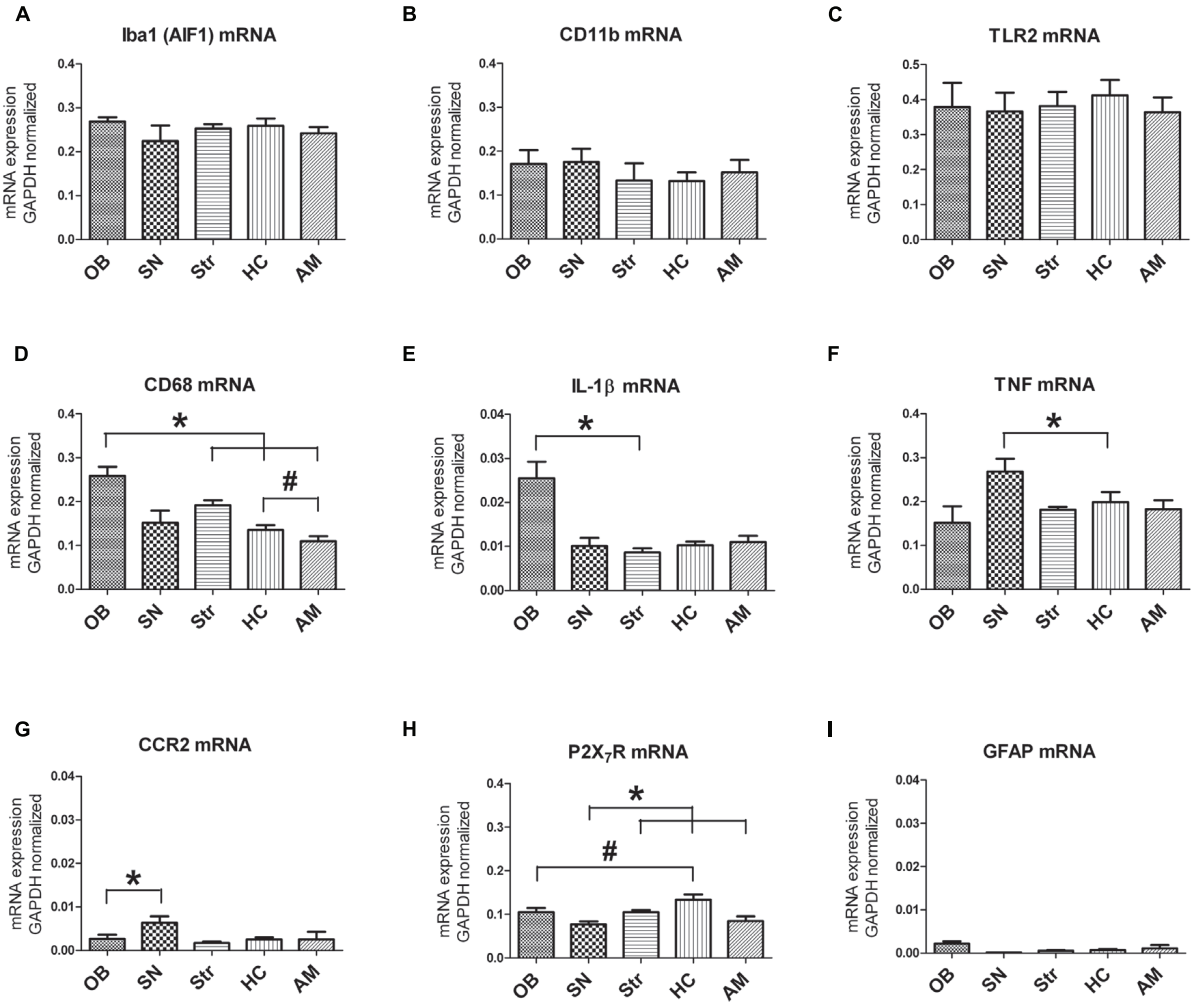
Region-specific expression levels of established microglial markers. No significant differences were found in AIF1 **(A)**, CD11b **(B)** and TLR2 **(C)** mRNA levels between the different brain regions. For CD68 **(D)** and IL-1β **(E)**, a significantly higher expression was present in the OB compared to other brain regions (CD68: OB vs. Str, HC, AM ^∗^*p* < 0.05; IL-1β: OB vs. Str ^∗^*p* < 0.05), and a significantly higher expression of CD68 (d) in Str compared to AM was found (^#^*p* < 0.02). Significantly higher TNF mRNA level **(F)** was measured in the SN relative to the HC (^∗^*p* < 0.05), a significantly higher CCR2 mRNA level **(G)** in SN compared to OB (^∗^*p* < 0.05), significantly lower P2X_7_R mRNA level **(H)** in SN compared to Str, HC, and AM (^∗^*p* < 0.05), and significantly more P2X_7_R expression **(H)** in the HC compared to OB (^#^*p* < 0.05). **(I)** No GFAP expression was observed, indicating that the sorted microglia were not contaminated by astrocytes.

To determine brain region-specific microglial expression, microglia were sorted from the OB, SN, Str, HC, and AM, and gene expression within these samples was analyzed by means of qPCR. The genes chosen for analysis have been described to be expressed in microglia *in vivo* or *in vitro*, both under control and/or inflammatory conditions. We determined clear mRNA expression of CD11b (itgam; general microglia) and AIF-1 (iba1; general microglia), CD68 (microglial activation), TNF (pro-inflammatory cytokine), TLR2 (pathogen recognition), P2X purinoreceptor 7 (P2X_7_R; receptor for ATP), C-C chemokines receptor 2 (CCR2; monocyte chemotaxis) and Interleukin-1β (IL-1β; pro-inflammatory cytokine; **Figures 3**). Macrophage scavenger receptor (MSR-1), involved in alternative microglial activation and phagocytosis, brain-derived neurotrophic factor (BDNF) and inducible nitric oxide synthase (NOS-2) expression levels were below the detection limit (data not shown). Moreover, hardly any GFAP expression was observed, indicating the absence of astrocyte contamination in the sorted microglia (**Figures 3C**). Normalized mRNA expression of the general microglial markers AIF-1 and CD11b showed no significant differences between the different brain regions studied (**Figures 3A,B**). There was a trend toward higher CD11b mRNA expression level in the OB compared to Str, and in the SN compared to the HC (**Figures 3B**; *p* = 0.059; *p* = 0.062, respectively). The mRNA level of the microglial activation marker CD68 was significantly higher in the OB compared to the Str, HC, and AM (**Figures 3D**; ^∗^*p* = 0.030, ^∗^*p* = 0.049, ^∗^*p* = 0.020, respectively), and in the Str compared to the AM (**Figures 3D**; ^#^*p* = 0.014). Furthermore, trends were present toward higher CD68 mRNA levels in Str compared to HC (**Figures 3D**; *p* = 0.075) and in HC relative to AM (**Figures 3D**; *p* = 0.091).

Interleukin-1β mRNA expression was significantly elevated in the OB compared to Str (**Figures 3E**; ^∗^*p* = 0.034) and a trend was observed toward more expression in the OB compared to all other regions (**Figures 3E**; SN *p* = 0.074; HC *p* = 0.056; AM *p* = 0.063), while TNFα mRNA level was significantly higher in the SN compared to HC (**Figures 3F**; ^∗^*p* = 0.047) in addition to a trend toward more expression in the SN compared to OB and AM (**Figures 3F**; *p* = 0.078; *p* = 0.068, respectively). TLR2, involved in pathogen recognition, was not different between the different brain regions and showed a similar expression pattern as AIF-1 mRNA (**Figures 3G**). This in contrast to CCR2, a receptor for CCL2 involved in microglial recruitment and proliferation (Prins et al., 2014), that was significantly elevated in the SN compared to the OB (**Figures 3H**; ^∗^*p* = 0.027). P2X_7_R, a purinoreceptor for ATP, was expressed at significantly lower amounts in the SN compared to HC, Str and AM (**Figures 3I**; ^∗^*p* = 0.015; ^∗^*p* = 0.008; ^∗^*p* = 0.040, respectively) but significantly higher in the HC relative to the OB (**Figures 3I**; ^#^*p* = 0.26). Notably, all values indicated here are based on paired-sample *t*-test with a significance set at 0.05, they did not survive Bonferroni corrections for multiple testing (*p* ≤ 0.005), and therefore should be interpreted with care.

## Discussion

By optimizing protocols to isolate pure microglia from several gray matter regions implicated in PD, we could determine brain-region specific microglial gene expression profiles. We demonstrate that already under control conditions, gray matter-derived microglial cells express markers that are essential not only for regulating brain homeostasis, but also do so in a region-specific manner.

For the present study, region-specific microglial gene expression was determined in Wistar rats, because this rat strain will be used for subsequent PD-related studies. However, we cannot exclude that the results obtained thus far in Wistar rats may differ when other rat strains, e.g., inbred strains, will be used.

Our current isolation protocol differs from other studies that isolated microglia from adult brain using enzyme digestion in order to obtain cell suspensions (Melief et al., 2013; Orre et al., 2014). Since we avoided enzymatic digestion, this prevented alterations in the expression of surface antigens that are important for cell identification (Ford et al., 1996). Moreover, in the present study we directly analyzed freshly isolated microglia, which is more representative for the *in vivo* status of these cells at the time of isolation (Frank et al., 2006; Melief et al., 2012; Olah et al., 2012). Also the isolation procedure itself may influence the cell status, which is why all brain regions were dissected, and the cells isolated and processed immediately and all at the same time. The whole procedure was further performed on ice to avoid microglial activation as much as possible and biological triplicates were used to determine variability. Thus, when microglia isolated from one brain region show a different gene expression profile from microglia isolated from another region, such results should then reflect region-specific properties of the cells.

Fluorescent-activated cell sorting analysis revealed that indeed around 93% of all isolated cells were microglia, characterized by CD11b^high^/Cd45^pos^ expression relative to lymphocytes CD11b^low^/Cd45^pos^ expression, after gating on size and granularity and viability. The remaining cells consisted of lymphocytes. Hence, after sorting for CD11b^high^/CD45^pos^ expressing cells, a pure population of microglia was obtained. The number of microglial cells obtained from the various gray matter brain regions differed substantially with the lowest yield from the SN, and the highest from the OB. This difference can be attributed to the size of the areas excised from which cells were isolated (**Figures 1**) but was controlled for during qPCR analysis. In line with previous studies, enrichment and purity of brain microglia was confirmed at the mRNA level by studying the expression of established general microglial markers CD11b and Iba1 in all brain regions (Ito et al., 1998; Frank et al., 2006; Torres-Platas et al., 2014). Based on hardly detectable GFAP mRNA levels, astrocyte contamination of the FACS sorted cells was excluded. We further characterized regional specificity of microglia in the healthy brain by studying the expression of genes that have previously been reported in microglia, under control and/or inflammatory conditions (Vroon et al., 2007; Lucin and Wyss-Coray, 2009; Borrajo et al., 2014; Doorn et al., 2014b; Prins et al., 2014).

Under healthy conditions, microglia display a ramified phenotype that is generally characterized by a low expression of molecules like CD68 and MHCII, and by a low phagocytic activity (Aloisi, 2001; Perry et al., 2007; Hart et al., 2012). CD68 is a glycoprotein which binds to low density lipoprotein and is used as a marker for both activated, amoeboid microglia, as well as primed microglia (Holness and Simmons, 1993; Wong et al., 2005; Eggen et al., 2013). Its expression has furthermore been related to aging (Perry et al., 1993; Conde and Streit, 2006). Our results show that more CD68 expression is present in the OB compared to other brain regions, which suggests that an activated or primed state of microglia exists in the OB, as we also confirmed with IL-1β measures. It has been shown before in naïve mice that expression of activational markers, including MHCII, is relatively high in parenchymal microglia (Zhang et al., 2002) Together, this could be representative for the clear antigen-presenting function that microglial cells can have, and with the notion that microglia in the OB are already in a primed state under normal conditions. This suggests that OB-derived microglia may be more prone to priming than microglia derived from other gray matter brain regions, which in turn may lead to differential outcomes between different brain regions when confronted with pathological conditions, e.g., α-synuclein aggregation.

Similar to CD68, also the pro-inflammatory cytokine IL-1β was expressed at higher level in the OB relative to other regions. IL-1β is rapidly upregulated in response to various inflammatory triggers and exerts a wide range of biological and physiological effects on the immune, endocrine and nervous system (Rothwell, 1991; van Dam et al., 1992; Wang et al., 2008). Our results are in line with previous data demonstrating that IL-1β protein is abundantly present in the OB of rats (Lim and Brunjes, 1999). Of interest to note is that we have demonstrated, using knock-out mice that IL-1β contributes to microgliosis, i.e., elevated CD68 expression, in the OB, but not in the SN (Vroon et al., 2007). Our present data thus support the notion that expression of CD68 and IL-1β expression are related. The enhanced expression observed in the OB relative to other regions could be due to the important role of the OB in odor discrimination in rodents. In particular the close connections to the outside world may be of ultimate importance for the microglial cells to be in an easily alerted state to monitor and respond to possible changes in homeostasis.

In contrast to IL-1β and CD68, mRNA levels of TNFα were found to be increased in the SN and to be markedly low in the OB, relative to other brain regions. TNFα has been considered a possible key inflammatory regulator that can induce cytokine production and (Sriram et al., 2002; Kraft et al., 2009). In the CNS, microglia are the primary source of TNFα (Hanisch, 2002) and TNFα release by microglia has been implicated in neurotoxicity (Mogi et al., 1994; Taylor et al., 2005; Borrajo et al., 2014). Interestingly, a region-specific, but dual role for TNFα itself has been suggested, e.g., in the brain of MPTP treated mice. In this model, it was proposed that TNFα promotes neurodegeneration in the Str but has a protective role in the HC (Sriram et al., 2006). Here, whereas we did not find differences in baseline TNFα expression between HC and Str, high expression levels were present in the SN. This increase in TNFα expression in the SN under normal conditions may underlie the increased neuronal vulnerability seen in the SN following additional challenges, such as during the development of α-synuclein pathology (Watson et al., 2012). A protein typically known to be involved in oxidative cell stress responses is NOS-2. Since we could not detect NOS-2 in microglia derived from various brain regions, this supports the notion that the naïve rats we used did not experience cell stress, and that experimental handling conditions of our samples did not induce oxidative stress in the cells either.

To further characterize microglial cell differences between different brain regions, we investigated their expression levels of several receptors. TLR2, a receptor for pathogen recognition, was recently implicated in the activation of microglia by α-synuclein *in vitro* (Beraud et al., 2011; Kim et al., 2013) and is thought to stimulate secretion of pro-inflammatory cytokines. Moreover, in tissue of PD patients, differences in TLR2 expression were found in microglia by us between the HC and SN of PD patients (Doorn et al., 2014b). Also overexpression of α-synuclein in rodents was reported to increase TLR2 mRNA specifically in the SN but not in other regions (Watson et al., 2012). However, in our current study using naïve rats, no differences were present in microglial TLR2 mRNA levels between the regions studied. This supports the idea that TLR2 is distributed in an evenly manner throughout the brain under normal conditions, and is mainly upregulated by microglia when they encounter specific pathogens, like α-synuclein, and as such, does not represent a marker for primed microglia.

Clear differences were found in CCR2 mRNA expression levels that were higher in the SN than in other gray matter regions. CCR2 is implicated in leukocyte migration and has recently been shown to be expressed by gray matter microglial cells and to be involved in cell proliferation (Hinojosa et al., 2011; Prins et al., 2014). In agreement, CCR2 deficiency markedly impaired microglial recruitment, accelerated β-amyloid accumulation and disease progression in a mouse model for Alzheimer’s disease (AD; El Khoury et al., 2007). Hence, the higher expression of CCR2 mRNA already at basal conditions in the SN could similarly be beneficial for microglial recruitment when homeostasis becomes disturbed. In contrast to AD, microglial recruitment in the SN is often associated with an aversive environment that could promote DA-ergic vulnerability to cell death in PD (Kim et al., 2000). The related high expression of TNFα in the SN (present study) thus supports an enhanced sensitivity of the SN to neurotoxicity.

As member of the purinergic receptor family, P2X_7_Rs are a class of ligand-gated ion channels activated by ATP that contributes to neuro- and glio-transmission. P2X_7_Rs are expressed by both neurons and glia in various brain regions and have been implicated in a wide variety of behaviors, including learning and memory (Chu et al., 2010; Burnstock et al., 2011) These receptors have also been described to be involved in responses to trauma, neurodegeneration, neuropsychiatric disorders and neuropathic pain (McLarnon et al., 2006; Burnstock et al., 2011; Carmo et al., 2014). Especially, the fast purinergic synaptic transmission that is in part mediated through these receptors, has been clearly identified (Pankratov et al., 2009) in a number of brain areas, including the HC (Mori et al., 2001; Pankratov et al., 2002). The higher P2X_7_R mRNA in the HC compared to other regions as observed in the present study could contribute to its role in regulating synaptic plasticity and memory formation in the HC (Wieraszko, 1996; Campos et al., 2014); this is likely an indirect effect via microglial cells (Chu et al., 2010). Alternatively, P2X_7_R on microglial cells are implicated in ATP-mediated microglial activation and cytokine release, of importance for brain disorders (Ni et al., 2013; Shieh et al., 2014). In addition, ATP-P2X_7_R signaling is involved in glial cell proliferation (Zou et al., 2012) which is apparent in the HC of presymptomatic PD patients (Doorn et al., 2014a). Thus, P2X_7_R in the HC has strategic implications for monitoring deviations in ATP levels resulting in cell proliferation and plasticity changes that likely contribute to adaptive behavior seen under disease conditions like PD.

Based on transcriptional profiling, proteomics and functional assays, microglia are typically classified into ‘classically’ activated microglia (M1 type) or ‘alternatively’ activated microglia (M2 type; Mantovani et al., 2004; Mosser and Edwards, 2008; Gordon and Martinez, 2010). M1 microglia are polarized by IFN-γ and then produce pro-inflammatory cytokines (e.g., IL-12, IL-1β, TNFα) and oxidative metabolites that can cause cytotoxicity. In contrast, IL-4, IL-10, and IL-13 polarize M2 microglia, which then downregulate pro-inflammatory cytokines, increase their expression of anti-inflammatory molecules and facilitate wound healing and repair, also within the damaged CNS (Mantovani et al., 2002; Kigerl et al., 2009; Gordon and Martinez, 2010; David and Kroner, 2011; Doorn et al., 2012). MSR1 and BDNF are proteins expressed by alternative activation of microglia and are related to growth factors, anti-inflammatory cytokine production and neuroprotection (Mantovani et al., 2002; Lucin and Wyss-Coray, 2009). The fact that these sets of genes were undetectable in our current study, whereas genes related to the classical activation state of microglia, e.g., CD68, IL-1β and TNFα were present, suggests that the brain region-specific differences in microglia gene expression profile under healthy conditions are more related to the classical activation pathway in these cells. This implies that genes expressed by alternatively activated microglia would become more important when homeostasis is derailed, but would remain undetectable under naïve conditions.

## Conclusion

The present data demonstrate that a unique basal gene expression signature of microglia exists that differs between different gray matter brain regions of Wistar rats. Within the local brain microenvironment, glial cells play critical roles in the homeostatic mechanisms that support neuronal survival. Since the basal profile of microglia is more tuned toward a pro-inflammatory profile and in a region-dependent way, such region-specific gene expression profiles of microglia likely predict different outcomes under challenging and disease conditions. As such, region-specific differences in neuronal susceptibility in conditions like PD, could at least in part, be attributable to basal differences in levels of inflammation-related receptors and other factors that are endogenously, and differentially, produced by microglial cells.

## Author Contributions

AvD and PL designed research; KD and JB performed research; IH and HB contributed analytic tools and protocols; KD, JB, BD, and AvD analyzed data; and KD, BD, AvD, and PL wrote the paper.

## ABBREVIATIONS

α-synuclein: alpha-synuclein
Aβ: amyloid-beta
AON: anterior olfactory nucleus
CA: cornus ammonis
GFAP: glial fibrillary acidic protein
HC: hippocampus
HPtau: hyperphosphorylated tau
Iba1: ionized calcium binding adaptor molecule 1
IL: interleukin
iLBD: incidental Lewy body disease
IR: immunoreactivity
LBs: Lewy bodies
LNs: Lewy neurites
MPTP: 1-methyl-4-phenyl-1,2,3,6-tetrahydropyridine
NFT: neurofibrillary tangles
OB: olfactory bulb
PD: Parkinson’s disease
SN: substantia nigra
TLR: toll like receptor
TNFα: tumor necrosis factor-alpha
